# PlncRNA-HDeep: plant long noncoding RNA prediction using hybrid deep learning based on two encoding styles

**DOI:** 10.1186/s12859-020-03870-2

**Published:** 2021-05-12

**Authors:** Jun Meng, Qiang Kang, Zheng Chang, Yushi Luan

**Affiliations:** 1 School of Computer Science and Technology, Dalian University of Technology, Dalian, 116024 Liaoning China; 2 School of Bioengineering, Dalian University of Technology, Dalian, 116024 Liaoning China

**Keywords:** Deep learning, Long short-term memory, Convolutional neural network, Plant, lncRNA, Prediction

## Abstract

**Background:**

Long noncoding RNAs (lncRNAs) play an important role in regulating biological activities and their prediction is significant for exploring biological processes. Long short-term memory (LSTM) and convolutional neural network (CNN) can automatically extract and learn the abstract information from the encoded RNA sequences to avoid complex feature engineering. An ensemble model learns the information from multiple perspectives and shows better performance than a single model. It is feasible and interesting that the RNA sequence is considered as sentence and image to train LSTM and CNN respectively, and then the trained models are hybridized to predict lncRNAs. Up to present, there are various predictors for lncRNAs, but few of them are proposed for plant. A reliable and powerful predictor for plant lncRNAs is necessary.

**Results:**

To boost the performance of predicting lncRNAs, this paper proposes a hybrid deep learning model based on two encoding styles (PlncRNA-HDeep), which does not require prior knowledge and only uses RNA sequences to train the models for predicting plant lncRNAs. It not only learns the diversified information from RNA sequences encoded by *p*-nucleotide and one-hot encodings, but also takes advantages of lncRNA-LSTM proposed in our previous study and CNN. The parameters are adjusted and three hybrid strategies are tested to maximize its performance. Experiment results show that PlncRNA-HDeep is more effective than lncRNA-LSTM and CNN and obtains 97.9% sensitivity, 95.1% precision, 96.5% accuracy and 96.5% F1 score on *Zea mays* dataset which are better than those of several shallow machine learning methods (support vector machine, random forest, k-nearest neighbor, decision tree, naive Bayes and logistic regression) and some existing tools (CNCI, PLEK, CPC2, LncADeep and lncRNAnet).

**Conclusions:**

PlncRNA-HDeep is feasible and obtains the credible predictive results. It may also provide valuable references for other related research.

## Backgroud

Noncoding RNAs (ncRNAs) are considered as non-protein-coding transcripts [1]. Long ncRNAs (lncRNAs) usually refer to the ncRNAs with longer than 200 nucleotides [2] and they play an important role in regulating biological activities [3]. For example, lncRNAs are players in cardiovascular diseases and atherosclerosis and they have attracted attention in cancer research [4, 5]. They are involved in the vernalization-mediated *FLOWERING LOCUS C* repression, which affects the flowering in *Arabidopsis* [6, 7]. lncRNAs are pivotal players on the regulation in a range of developmental processes in plant [3, 8]. A growing number of plant lncRNAs have been gradually discovered, but their diverse functions are not appreciated enough. The prediction of plant lncRNAs is important for exploring the functional lncRNAs expressed in genomes and understanding their mechanisms.

Bioinformatics technology has been widely used in biological prediction. The traditional methods often use the physicochemical, sequential and structural features (codon frequency [9], open reading frame (ORF) [10] and similarity of known proteins [11]) as the inputs to train a shallow machine learning model (support vector machine (SVM) [12], random forest (RF) [13], k-nearest neighbor (k-NN) [14], etc.) for prediction. CNCI is a powerful tool, and it uses adjoining nucleotide triplets to train SVM for classifying protein-coding and noncoding sequences [15]. PLEK, an alignment-free tool, uses a computational pipeline based on improved *k*-mer and SVM to distinguish lncRNAs from messenger RNAs (mRNAs) [16]. CPC is a classification tool based on SVM, which uses the sequence features to classify coded and noncoding RNAs [17] and its new version CPC2 with faster speed and higher accuracy has been published [18]. With the development of computer technology, deep learning has showed better performance and adaptability than shallow machine learning in many fields [19]. It is an end-to-end learning, which extracts the potential features of the data and learns the rule by optimizing the loss function to avoid manually designing rule. LncADeep integrates intrinsic and homologous features into the deep belief network to construct models targeting full-length and partial-length transcripts for classifying lncRNAs [20]. lncRNAnet incorporates the recurrent neural network (RNN) for RNA sequence modeling and the convolutional neural network (CNN) for detecting stop codons to obtain an ORF indicator in lncRNA classification [21]. However, none of these studies avoids the complex feature engineering, which is not only a time-consuming process, but also requires the prior knowledge, such as a deep understanding of physicochemical, sequential and structural features of RNA and the proper use of some bioinformatics tools. It is significant to develop an efficient method that only uses RNA sequences to train the models and obtains credible predictive results.

In natural language processing and image classification, deep learning technology is used to automatically extract and learn abstract information from the data to train the model, which shows superior performance and strong adaptability and avoids complex feature engineering [19]. Inspired by it, the prediction of lncRNAs can be considered as natural language processing and image classification problems. Long short-term memory (LSTM) is an appropriate model that has been successfully applied to natural language processing [22]. The sentences in natural language can be converted into the vectors as input of LSTM for training. CNN is appropriate for image classification [23]. The image can be converted into the two-dimensional matrices as input of CNN for training. Furthermore, RNA sequences can be encoded into different forms as the inputs to train a variety of base models. The ensemble of them not only learns the information from multiple encoding forms, but also ensures the diversity of base models, and thus obtains better performance than a single model [24, 25]. Therefore, the raw RNA sequences can be encoded as vectors and matrices as the inputs to train LSTM and CNN respectively, and then the trained models are hybridized to comprehensively predict lncRNAs.

Up to now, various methods and tools for predicting animal lncRNAs have been published, while few for plant. Since ncRNAs are mainly transcribed by RNA polymerase II in animal and transcribed by RNA polymerases II, IV and V in plant [26], and plant lncRNAs have low level expression and cross-species conservation [27], the predictors for animal do not guarantee the reliability to plant. Facing with these challenges, it is urgent and necessary to construct a reliable and powerful predictor for plant lncRNAs.

In this paper, plant lncRNAs are predicted by using hybrid deep learning based on two encoding styles (PlncRNA-HDeep). K-means clustering [28] is used to solve the undersampling of negative sample in dataset. The raw RNA sequences are first encoded as vectors and matrices by *p*-nucleotide [29] and one-hot [30] encodings respectively. Then, the encoded sequences are input into lncRNA-LSTM proposed in our previous study [29] and CNN for training respectively. Finally, the trained models are hybridized at decision level to obtain the final predictive results. PlncRNA-HDeep only uses RNA sequences to train the models for predicting plant lncRNAs. It learns the diversified information from two encoding styles and takes advantages of lncRNA-LSTM and CNN. The value of *p* in *p*-nucleotide encoding is adjusted and three hybrid strategies are tested to maximize the performance. PlncRNA-HDeep is more effective than lncRNA-LSTM and CNN. It also obtains the best results on *Zea mays* dataset compared with the shallow machine learning methods, such as SVM, RF, k-NN, decision tree (DT), naive Bayes (NB) and logistic regression (LR), and the existing tools, such as CNCI, PLEK, CPC2, LncADeep and lncRNAnet.

## Results

### Effects of value of *p* and hybrid strategy variations

**Fig. 1:**
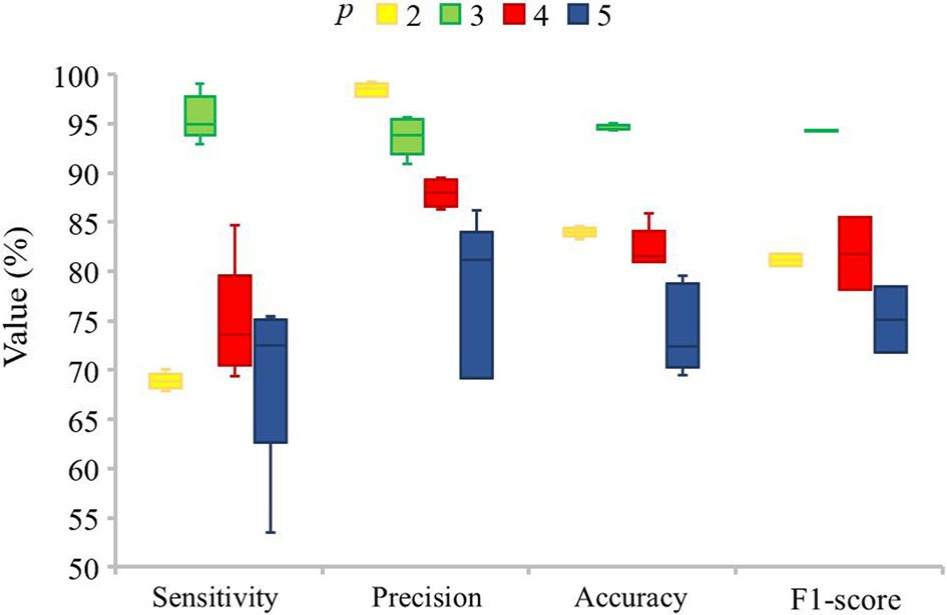
Effect evaluations of different values of *p* in lncRNA-LSTM using 5-fold cross validation

The value of *p* in *p*-nucleotide encoding is an important parameter that affects the performance of lncRNA-LSTM and thus the performance of PlncRNA-HDeep. 5-fold cross validation is used to evaluate the effects of different values of *p* in lncRNA-LSTM and the results are obtained (Fig. 1).

When *p* is 3, lncRNA-LSTM obtains the best sensitivity, accuracy and F1 score, its precision is the second best among all methods. Thus, the value of *p* is set to 3 in the follow experiments.

**Fig. 2:**
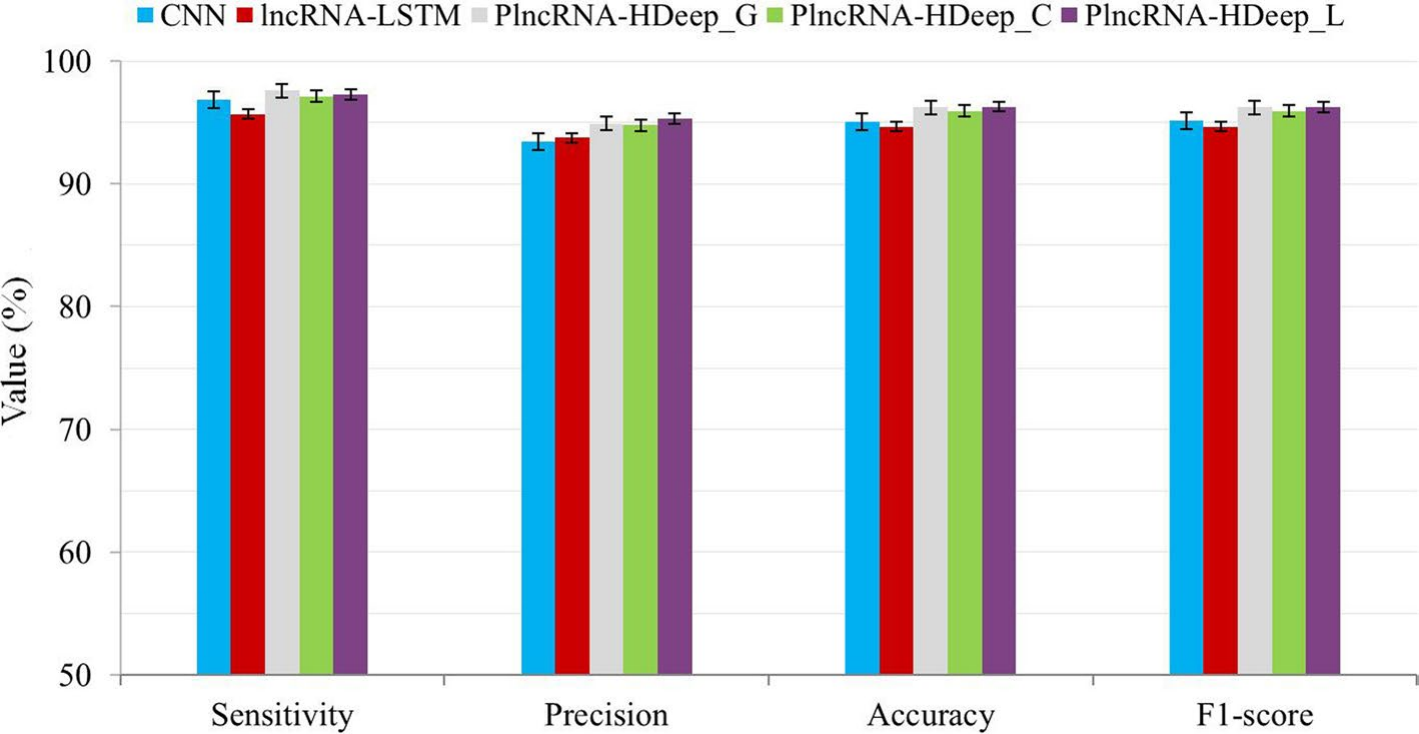
Effect evaluations of different hybrid strategies in PlncRNA-HDeep using 5-fold cross validation

The effects of different hybrid strategies in PlncRNA-HDeep are evaluated using 5-fold cross validation and the results are obtained (Fig. 2). Least significant difference (LSD) test is used to test statistically the accuracy of them and the significant difference is evaluated according to the obtained *p* value (Table 1).

**Table 1:** Least significant difference of compared methods

Method	Compared method	*p* value
PlncRNA-HDeep_G	CNN	0.001^+^
	lncRNA-LSTM	0.001^+^
	PlncRNA-HDeep_C	0.078^+^
	PlncRNA-HDeep_L	0.745^−^
PlncRNA-HDeep_C	CNN	0.001^+^
	lncRNA-LSTM	0.001^+^
	PlncRNA-HDeep_G	0.078^−^
	PlncRNA-HDeep_L	0.040^−^
PlncRNA-HDeep_L	CNN	0.001^+^
	lncRNA-LSTM	0.001^+^
	PlncRNA-HDeep_G	0.745^+^
	PlncRNA-HDeep_C	0.040^+^

“ + ” means the method obtains better accuracy than the compared method. “−” means the compared method obtains better accuracy than the method. There is significant difference between the results obtained by two methods with the significance level of 0.05 when *p* value ⩽ 0.05

PlncRNA-HDeep with different hybrid strategies always obtains better results than CNN and lncRNA-LSTM. It also shows the significant accuracy over CNN and lncRNA-LSTM with the significance level of 0.05 from LSD test results. It means that the three hybrid strategies are all effective for enhancing the performance of a single CNN and lncRNA-LSTM. The PlncRNA-HDeep methods with three hybrid strategies are compared with each other. PlncRNA-HDeep_G obtains the best sensitivity, and PlncRNA-HDeep_L obtains the best precision. They also obtain the similar accuracy and F1 score. PlncRNA-HDeep_C does not obtain the best result in each criterion. From LSD test results, PlncRNA-HDeep_L shows the significance on accuracy over PlncRNA-HDeep_C with the level of 0.05. Although PlncRNA-HDeep_G also obtains better accuracy than PlncRNA-HDeep_C, there is no significant difference between their results. Accordingly, PlncRNA-HDeep with the predominance of LSTM hybrid strategy (PlncRNA-HDeep_L) is selected in the following experiments.

### Impacts of balanced and imbalanced sample datasets

The number of negative sample may affect the performance of PlncRNA-HDeep [31]. The datasets with different ratios of positive samples and negative samples are used to verify the performance (Table 2).

**Table 2:** Impact evaluations of balanced and imbalanced sample datasets on performance of PlncRNA-HDeep

Ratio	F1-score (%)	AUC (%)	GM (%)
1:1	96.5	99.3	96.5
1:2	76.5	91.6	82.7
1:3	70.4	91.1	80.7

“Ratio” refers to the ratio of positive samples and negative samples in the dataset

On the imbalanced sample datasets, the performance of PlncRNA-HDeep is significantly degraded. Specially, on the imbalanced sample dataset with a ratio of positive samples and negative samples of 1:3, the F1 score, AUC and GM decrease 26.1%, 8.2% and 15.8% compared with them on the balanced sample dataset respectively. To ensure a good performance of PlncRNA-HDeep, the balanced sample dataset is finally adopted.

### Performance comparison with shallow machine learning methods

**Fig. 3:**
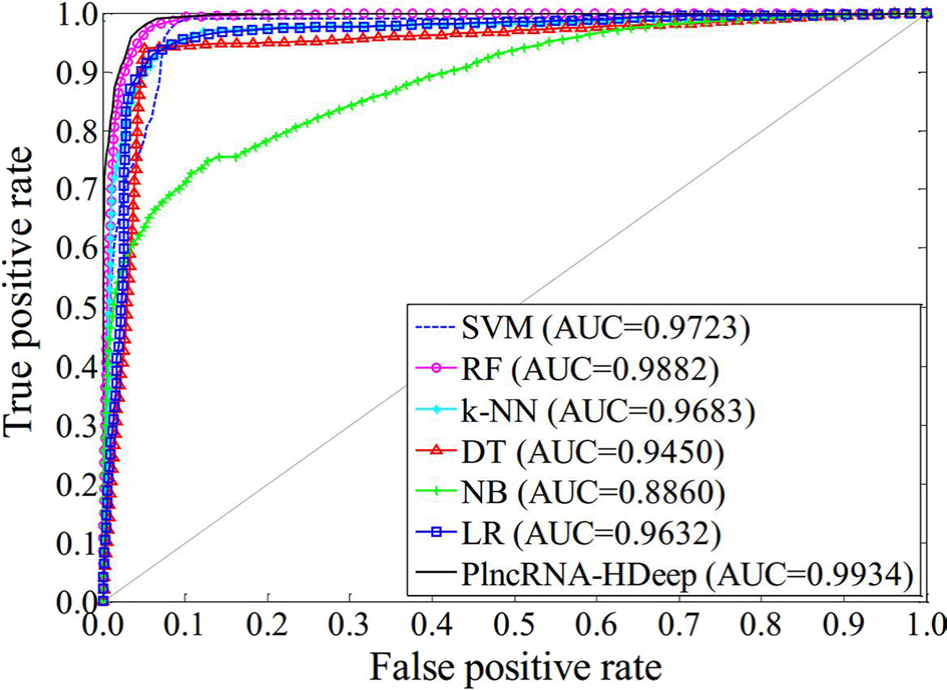
ROC curves and AUC values obtained by PlncRNA-HDeep and six shallow machine learning methods

To verify the performance of proposed model, PlncRNA-HDeep is compared with six shallow machine learning methods, which are SVM, RF, k-NN, DT, NB and LR (Table 3). Moreover, the ROC curves of them are plotted and the AUC values are obtained (Fig. 3).

**Table 3:** Performance of PlncRNA-HDeep compared with six shallow machine learning methods

Method	Sensitivity (%)	Precision (%)	Accuracy (%)	F1-score (%)
SVM	87.8	92.3	90.6	90.0
RF	95.2	95.1	95.3	95.1
k-NN	90.6	94.0	92.7	92.3
DT	93.9	94.6	94.5	94.3
NB	76.7	80.3	80.0	78.4
LR	84.4	96.4	91.0	90.0
PlncRNA-HDeep	97.9	95.1	96.5	96.5

PlncRNA-HDeep obtains 97.9% sensitivity, 95.1% precision, 96.5% accuracy and 96.5% F1 score. Its sensitivity, accuracy and F1 score are the best and precision is the second best among all methods. Its AUC achieves 0.9934 which is also better than those obtained by the other methods. RF obtains the second best sensitivity, precision, accuracy, F1 score and AUC, where precision is same as PlncRNA-HDeep’s. DT obtains the third best sensitivity, accuracy and F1 score, but its precision and AUC are not in top three of all methods. Although LR obtains the best precision, its other results are all not in top three. SVM obtains the third best AUC, but its other results are unsatisfactory. All results of k-NN and NB are not in top three, where NB’s results are the worst among all methods.

### Performance comparison with existing tools

To further verify the performance of PlncRNA-HDeep, it is compared with five existing tools (CNCI, PLEK, CPC2, LncADeep and lncRNAnet) which have been described in background section, and the results are obtained (Table 4).

**Table 4:** Performance of PlncRNA-HDeep compared with five existing tools

Tool	Sensitivity (%)	Precision (%)	Accuracy (%)	F1-score (%)
CNCI	64.5	90.5	78.9	75.3
PLEK	93.3	68.4	75.1	78.9
CPC2	88.4	91.9	90.3	90.1
LncADeep	66.6	91.0	80.0	76.9
lncRNAnet	72.0	73.3	72.9	72.6
PlncRNA-HDeep	97.9	95.1	96.5	96.5

All values obtained by PlncRNA-HDeep are the best compared with the other tools. Its accuracy is 17.6%, 21.4%, 6.2%, 16.5% and 23.6% better than that of CNCI, PLEK, CPC2, LncADeep and lncRNAnet respectively. The sensitivity and precision of PlncRNA-HDeep are 97.9% and 95.1% respectively and the difference of them is 2.8%, which shows good robustness of PlncRNA-HDeep. CPC2 obtains the second best accuracy and the difference between its sensitivity and precision is 3.5%. The accuracies of CNCI, PLEK and LncADeep achieve 75% but not more than 80%. The sensitivity of CNCI and LncADeep are about 25% worse than the precision of them respectively, which indicates that they tend to predict lncRNA as the negative sample. The sensitivity of PLEK is obviously better than the precision of it, which indicates that it tends to predict mRNA as lncRNA. The difference between the sensitivity and precision of lncRNAnet is 1.3%, which shows the best robustness. However, its accuracy does not achieve 75%.

## Discussion

lncRNA-LSTM with *p* = 3 in *p*-nucleotide encoding obtains the best results, which means that when every three continuous nucleotides in RNA sequence are regarded as a word, the sample can be better characterized. For the negative samples (mRNAs), this may due to every three continuous nucleotides determine a codon, which further determines the amino acid [32]. For the positive samples (lncRNAs), this may due to that the conservative triplet codon characteristics are needed to perform their functions, such as matching the interacted protein sequence [9]. From another perspective, when the value of *p* is 1 or 2, each sample can only be encoded by 5 or 17 integers (including zero-padding), which is not enough to characterize the sample, especially for lncRNA with longer than 200 nucleotides. When the value of *p* is more than 3, the sample length is greatly shortened after encoding, and the information that can be extracted is limited, which is not conducive to model training.

PlncRNA-HDeep with the predominance of LSTM hybrid strategy obtains the best results, which means that lncRNA-LSTM is used as the main model and CNN is used to assist in prediction. On the one hand, lncRNA-LSTM is an improved model that it is more suitable as the main model than the basic CNN [29]. On the other hand, *p*-nucleotide encoding characterizes the sample with a variety of integers, while one-hot encoding characterizes the sample with a 0–1 matrix, thus lncRNA-LSTM learns more information from the sample than CNN to show better performance.

In view of the successful application of LSTM and CNN in natural language processing and image processing respectively, the RNA sequences are encoded into vectors and matrices to train lncRNA-LSTM and CNN respectively [22, 23]. It takes advantage of the two deep learning models and further enhances the performance through hybridization [24, 25]. Therefore, PlncRNA-HDeep performs better than a single deep learning or shallow machine learning model. Since lncRNAs are different in animal and plant, the predictors for animal do not guarantee the reliability to plant [26]. It is conceivable that the plant predictor PlncRNA-HDeep obtains better results than other tools on plant lncRNA prediction. In addition, PlncRNA-HDeep only needs to input RNA sequences to complete training and prediction, which is simple and friendly for users. As a representative species, *Zea mays* is widely cultivated in the world. PlncRNA-HDeep has a good performance on *Zea mays* dataset, which indicates that it has potential to be applied to many other plant species.

## Conclusions

In this paper, a hybrid deep learning using two encoding styles, PlncRNA-HDeep, is presented to predict plant lncRNAs. It encodes the sample sequences using *p*-nucleotide and one-hot encodings for training lncRNA-LSTM and CNN respectively, and hybridizes the two models at decision level. It only uses the RNA sequences as the inputs to learn diversified information and takes advantages of lncRNA-LSTM and CNN. The performance of PlncRNA-HDeep is verified by comparing with the shallow machine learning methods, including SVM, RF, k-NN, DT, NB and LR, and the existing tools, including CNCI, PLEK, CPC2, LncADeep and lncRNAnet. The experiment results show that PlncRNA-HDeep is quite an efficient method. It may also provide valuable references for other related studies.

The future work will try to implement PlncRNA-HDeep for using online or downloading free. As the research progresses, the public databases of plant will become more abundant and more lncRNAs will be published. The widely application of PlncRNA-HDeep is also worth expecting.

## Methods

### Datasets

*Zea mays* is a kind of model plant which is widely used as research subject and has an important research significance. To train the deep learning model adequately and avoiding under-fitting, a large amount of published lncRNA data of *Zea mays* with abundant genetic annotation information were selected. 18,110 validated lncRNA sequences were downloaded from Green noncoding database (GreeNC) v1.12 [33] as the positive samples. 18,000 samples of them were selected randomly to generate a positive dataset.

From RefSeq database (https://www.ncbi.nlm.nih.gov/refseq/), 57,776 mRNA sequences were downloaded, the repeated sequences were filtered out, and 54,282 sequences were obtained as the negative samples. To generate a balanced sample dataset, the negative samples were undersampled. *k*-mer frequency of each negative sample sequence was extracted [9]. K-means, an unsupervised clustering method [28], was used to cluster these negative samples based on their *k*-mer frequencies. *k* was set to 1 and 2 and the clustering center point was set to 200 to save time and reduce the computational complexity. The number of samples in each cluster was recorded as *x*_*i*_ (*i* = 1, 2, …, 200). *O*_*i*_ (*i* = 1, 2, …, 200) samples were selected randomly from the *i*-th cluster as follows:

1 $$O_{i} = round\left( {\frac{{x_{i} }}{54282} \times {18000}} \right)$$

where *round*() is the rounded function. The 18,000 selected samples were used to generate a negative dataset. Other two imbalance sample datasets were also generated using the above method, where the positive dataset kept 18,000 samples and the ratios of positive samples and negative samples were 1:2 and 1:3 respectively [31].

80% of the samples from the positive and negative datasets were selected randomly for training and validation, and the other 20% of the samples were tested.

### Two encoding styles

**Fig. 4:**
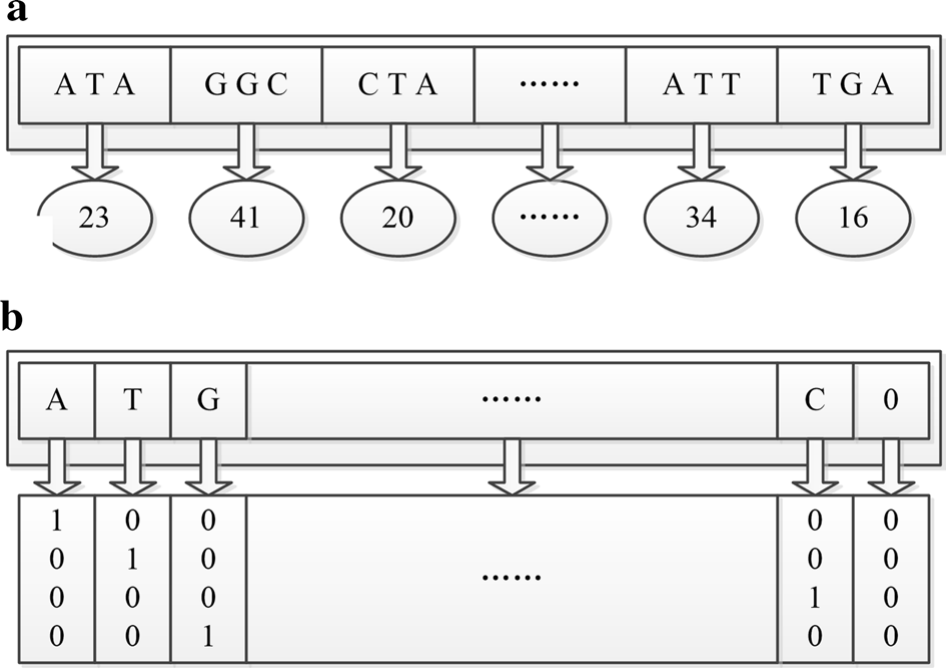
Two encoding styles. **a** *p*-nucleotide encoding when the value of *p* is 3. **b** one-hot encoding

Word segmentation is an important step in natural language processing and it encodes a sentence into a number vector [34]. Each RNA sequence is composed of nucleotide permutations, which is considered as a sentence. Thus, it can be encoded by “word segmentation” according to its biological characteristics. In the datasets, each sample was a chain-like molecule and composed by four bases (A, T, C and G) [35]. Each of the continuous *p* nucleotides (*p*-nucleotide) in RNA sequence was regard as a “word”. The value of *p* could be 2, 3, 4, ..., which corresponded to 16, 64, 256, … *p*-nucleotide formats respectively. Each *p*-nucleotide format is represented by a unique positive integer from 1 to 4^*p*^. A window with both length and step size of *p* slid along the RNA sequence to encode each *p*-nucleotide format into a corresponding positive integer. To ensure that all samples have the same length after encoding, the samples with a length less than the longest one are zero-padded. Then each sample is encoded into a number vector (Fig. 4a).

One-hot is a common encoding style [30]. Here the rule of one-hot encoding is set to that, A is encoded as (1, 0, 0, 0)^T^, T is encoded as (0, 1, 0, 0)^T^, C is encoded as (0, 0, 1, 0)^T^ and G is encoded as (0, 0, 0, 1)^T^. Then each sample sequence is encoded into a 0–1 matrix (similar as a two-dimensional grayscale image) of four rows and *N* columns, where *N* is set to the sequence length of the longest one among all samples. For those samples whose sequence length is less than *N*, the zero-padding is performed on their empty columns (Fig. 4b).

### Feature extraction of RNAs

*k*-mer frequency is the common sequence feature of RNAs [9]. For a sample consisting of A, T, C and G, a *k*-mer contains *k* continuous bases to generate 4^*k*^ different forms. If the value of *k* is too large, it increases the training and test time, and leads to many zeros in the feature vector to adversely affect the model training. The *k*-mer frequency with a large proportion also affects the role of other types of feature in model training. Therefore, 1-mer, 2-mers and 3-mers frequencies were extracted. A sliding window of length *k* was used to match *k*-mer along the sequence, the sliding step size was set to 1, and the frequency *f*_*j*_ was recorded as follows:

2 $$s_{k} = L - k + 1,\quad k = 1,2,3$$

3 $$a_{k} = \frac{1}{{4^{3 - k} }},\quad k = 1,2,3$$

4 $$f_{j} = a_{k} \frac{{c_{j} }}{{s_{k} }},\quad k = 1,2,3,\quad j = 1,2,...,84$$

where *s*_*k*_ is the total number of matches, *L* is the length of the RNA sequence, *a*_*k*_ is a parameter to make each *k*-mer frequency has the same effect, *c*_*j*_ is the number of matches of the *j*-th form.

ORF is a segment of the RNA sequence that has the potential translation ability. The ORF coverage rate of mRNA is significantly higher than that of lncRNA [10]. The ORF information of each sample was obtained by TransDecoder v3.0.1 (https://github.com/TransDecoder/TransDecoder), and the integrity (int), coverage (cov) and normalized ORF (nORF) were extracted as follows:

5 $${\text{int}} = \left\{ {\begin{array}{*{20}l} {0,} \hfill & {{\text{there}}\,{\text{is}}\,{\text{no}}\,{\text{integrated}}\,{\text{ORF}}} \hfill \\ {1,} \hfill & {{\text{there}}\,{\text{is}}\,{\text{integrated}}\,{\text{ORF}}} \hfill \\ \end{array} } \right.$$

6 $${\text{cov}} = \frac{{\sum\nolimits_{m = 1}^{n} {l_{m} } }}{L}$$

7 $${\text{nORF}} = \frac{n}{L}$$

where *n* is the number of ORF, *l*_*m*_ is the length of the *m*-th ORF.

Structure of RNA forms an important intermediate level of description of nucleic acids. The stability of the structure is related to the number of base pairs in the sequence and GC content. The more stable the structure, the more free energy it releases. The structure information of each sample was obtained by RNAfold in ViennaRNA Package v2.4.11 [11], and the number of base pairs, GC content (GCcont) and normalized minimum free energy (nMFE) were extracted as follows:

8 $${\text{GCcont}} = \frac{{{\text{NG}} + {\text{NC}}}}{{{\text{NA}} + {\text{NT}} + {\text{NC}} + {\text{NG}}}}$$

9 $${\text{nMFE}} = \frac{{\text{MFE}}}{L}$$

where NA, NT, NC and NG are the number of A, T, C and G in a sample respectively, MFE is the minimum free energy.

All extracted features were combined into a 90-dimensional vector as input for shallow machine learning methods in the comparison experiment. The extracted 1-mer and 2-mers frequencies were also used for clustering the negative samples in the creation of the datasets.

### Architectures of lncRNA-LSTM and CNN

LSTM is a kind of RNN with gated structure [36]. Bidirectional LSTM is a further extension to solve the problem that LSTM only processes single direction information. It extracts information to update the network from both the positive and negative directions as follows:

10 $${\mathop{h}\limits^{\rightarrow}}_{t} = \sigma \left( {W_{{x{\mathop{h}\limits^{\rightarrow}}_{t} }} x_{t} + W_{{{\mathop{h}\limits^{\rightarrow}} {\mathop{h}\limits^{\rightarrow}} }} {\mathop{h}\limits^{\rightarrow}}_{t - 1} + b_{{{\mathop{h}\limits^{\rightarrow}} }} } \right)$$

11 $${\mathop{h}\limits^{\leftarrow}}_{t} = \sigma \left( {W_{{x{\mathop{h}\limits^{\leftarrow}}_{t} }} x_{t} + W_{{{\mathop{h}\limits^{\leftarrow}}{\mathop{h}\limits^{\leftarrow}} }} {\mathop{h}\limits^{\leftarrow}}_{t - 1} + b_{{{\mathop{h}\limits^{\leftarrow}} }} } \right)$$

where *σ*() is the sigmoid function, *h* is the vector in the hidden layer, “→” and “←” are the positive and negative directions respectively, *t* is the time, *W* is the weight, *x* is the input, *b* is the bias. The output of the two networks is superimposed as follows:

12 $$y_{t} = W_{{{\mathop{h}\limits^{\rightarrow}} y}} {\mathop{h}\limits^{\rightarrow}}_{t} + W_{{{\mathop{h}\limits^{\leftarrow}} y}} {\mathop{h}\limits^{\leftarrow}}_{t} + b_{y}$$

where *y* is the output.

**Fig. 5:**
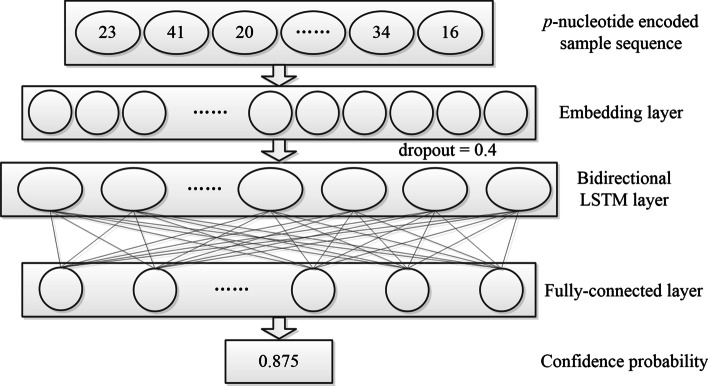
Architecture of lncRNA-LSTM

lncRNA-LSTM is a LSTM-based model constructed in our previous study [29]. Its architecture contains a word embedding layer, a bidirectional LSTM layer and a fully-connected layer. In the bidirectional LSTM layer, the units was set to 64 and the dropout rate was set to 0.4. In the fully-connected layer, “sigmoid” was selected as the activation function. The binary cross entropy loss function was selected to calculate the loss which was optimized by using the “Adam” optimizer. The parameters of each layer were updated by backpropagation. Each *p*-nucleotide encoded sample sequence was input as a 4^*p*^-dimensional vector into lncRNA-LSTM. Different from the overview of lncRNA-LSTM in [29], here the output was mapped to [0, 1] interval to obtain the confidence probability instead of the label. Its value indicated the confidence that the corresponding sample was predicted as a lncRNA (Fig. 5).

CNN is a popular deep learning model, a basic CNN structure usually includes the convolutional layer, pooling layer and fully-connected layer [19]. The convolutional layer outputs feature maps by convolving the feature maps of the previous layer with a set of filters as follows:

13 $$Fm_{out} = \sum\limits_{j = 1}^{Nf} {Ft_{j} \times Fm_{in} + b}$$

where *Fm*_*out*_ is the output feature maps, *Fm*_*in*_ is the input feature maps, *Ft*_*j*_ means the *j*-th filter, *Nf* is the number of filters, *b* is the bias. The pooling layer combines the outputs of one layer of neuron clusters into a single neuron in the next layer, and the commonly used schemes are max-pooling and average-pooling. The fully-connected layer connects every neuron in one layer to every neuron in another layer.

**Fig. 6:**
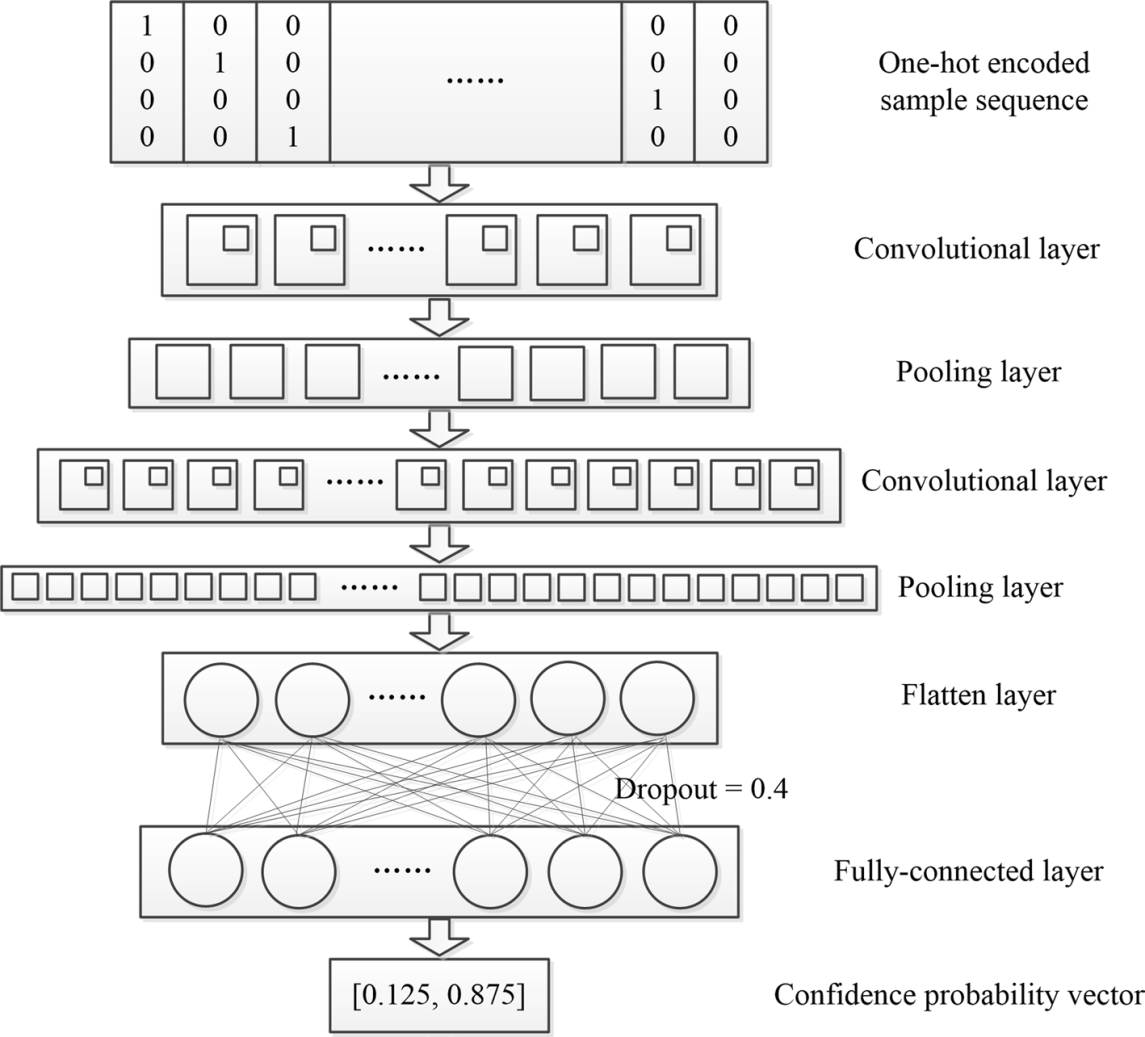
Architecture of CNN

The architecture of CNN in this paper was mainly constructed by two convolutional layers, two pooling layers and a fully-connected layer. In the convolutional layers, the number of filters were set to 32 and 64 respectively. In the pooling layers, the max-pooling schemes were used. In the fully-connected layer, the dropout rate was set to 0.4 and “softmax” was selected as the activation function. The categorical cross entropy loss function was selected to calculated the loss which was optimized by using the “SGD” optimizer. The parameters of each layer were updated by backpropagation. All parameter selections were referred to the related studies [37] and our previous experiences [38]. Each one-hot encoded sample sequence was input as a 4 * *N* matrix into above CNN. The output was mapped to [0, 1] interval to obtain a 2-dimensional confidence probability vector. The values of this vector indicated the confidence that the corresponding sample was predicted as mRNA and lncRNA respectively (Fig. 6).

### Hybrid deep learning

lncRNA-LSTM and CNN were trained respectively, and used to predict the input sample sequence to output the confidence probabilities. Then they were hybridized on decision level based on three hybrid strategies.

The first was the greedy hybrid strategy (the method is denoted as PlncRNA-HDeep_G), which was inspired by greedy selection [39]. It always selected the higher one of the two confidence probabilities obtained by two models respectively as follows:

14 $$Cp{ = }\left\{ {\begin{array}{*{20}l} {Cp_{C} ,} \hfill & {abs\left( {2Cp_{L} - 1} \right) \le abs\left( {2Cp_{C} - 1} \right)} \hfill \\ {Cp_{L} ,} \hfill & {{\text{other}}} \hfill \\ \end{array} } \right.$$

where *abs*() is the absolute value function, *Cp* is the confidence probability that the sample is predicted as a lncRNA, *Cp*_*L*_ and *Cp*_*C*_ are *Cp* obtained by lncRNA-LSTM and CNN respectively.

The second was the predominance of CNN hybrid strategy (the method was denoted as PlncRNA-HDeep_C). It selected the confidence probability obtained by CNN. However, when this confidence probability was not high enough, it selected the confidence probability obtained by lncRNA-LSTM as follows:

15 $$Cp = \left\{ {\begin{array}{*{20}l} {Cp_{L} ,} \hfill & {abs\left( {2Cp_{C} - 1} \right) \le 0.5} \hfill \\ {Cp_{C} ,} \hfill & {{\text{other}}} \hfill \\ \end{array} } \right.$$

The third was the predominance of LSTM hybrid strategy (the method was denoted as PlncRNA-HDeep_L). It was similar as the predominance of CNN hybrid strategy except that CNN and lncRNA-LSTM were exchanged as follows:

16 $$Cp = \left\{ {\begin{array}{*{20}l} {Cp_{C} ,} \hfill & {abs\left( {2Cp_{L} - 1} \right) \le 0.5} \hfill \\ {Cp_{L} ,} \hfill & {{\text{other}}} \hfill \\ \end{array} } \right.$$

The final obtained confidence probability *Cp* was mapped to [0, 1] interval. The label, as the output of the hybrid deep learning, could be 1 (when *Cp* ≥ 0.5) or 0 (when *Cp* < 0.5), which indicated the corresponding sample was predicted as lncRNA or not respectively.

### Implement of PlncRNA-HDeep

PlncRNA-HDeep was implemented by Keras 2.2.4 and all parameters used the default values from Keras documentation (https://keras.io/). All scripts were written by Python 3.6.5. The whole project was implemented on PC with 2.81 GHz CPU, 6 GB GPU and 8 GB RAM memory under a Microsoft Windows 10 operating system.

### Evaluation criteria

The performance evaluation criteria in the experiments are as follows:

17 $${\text{Sensitivity}} = \frac{{{\text{TP}}}}{{{\text{TP}} + {\text{FN}}}}$$

18 $${\text{Precision}} = \frac{{{\text{TP}}}}{{{\text{TP}} + {\text{FP}}}}$$

19 $${\text{Accuracy}} = \frac{{{\text{TP}} + {\text{TN}}}}{{{\text{TP}} + {\text{TN}} + {\text{FP}} + {\text{FN}}}}$$

20 $${\text{F1-score}} = \frac{{{\text{2TP}}}}{{{\text{2TP}} + {\text{FP}} + {\text{FN}}}}$$

21 $${\text{GM}} = \sqrt {\frac{{{\text{TP}}}}{{{\text{TP}} + {\text{FN}}}} \times \frac{{{\text{TN}}}}{{{\text{TN}} + {\text{FP}}}}}$$

where true positive (TP) refers to the number of true lncRNAs which are correctly predicted, false negative (FN) refers to the number of true lncRNAs which are incorrectly predicted as mRNAs, false positive (FP) refers to the number of true mRNAs which are incorrectly predicted as lncRNAs, true negative (TN) refers to the number of true mRNAs which are correctly predicted. Sensitivity is the percentage of the correctly predicted lncRNAs in all true lncRNAs. Precision is the percentage of the correctly predicted lncRNAs in all samples predicted as lncRNAs. Accuracy is the percentage of the correctly predicted samples in the total samples. F1 score (F1-score) is a harmonic average of sensitivity and precision. Geometric mean (GM) is a common criterion that gives a more accurate evaluation on imbalanced sample dataset. In addition, area under curve (AUC) from receiver operating characteristic (ROC) curve is also used for evaluation. The value of AUC ranges from 0 to 1, where AUC = 1 stands for the perfect prediction.

## Data Availability

The source code of PlncRNA-HDeep and the used dataset are available at https://github.com/kangzhai/PlncRNA-HDeep.

## Abbreviations

CNN: Convolutional neural network
DT: Decision tree
GreeNC: Green noncoding database
k-NN: k-Nearest neighbor
lncRNA: Long noncoding RNA
LR: Logistic regression
LSD: Least significant difference
LSTM: Long short-term memory
mRNA: Messenger RNA
NB: Naive Bayes
ORF: Open reading frame
RF: Random forest
RNN: Recurrent neural network
SVM: Support vector machine
